# Statin use and the risk of ovarian and endometrial cancers: a meta-analysis

**DOI:** 10.1186/s12885-019-5954-0

**Published:** 2019-07-24

**Authors:** Yizi Wang, Fang Ren, Zixuan Song, Peng Chen, Shuang Liu, Ling Ouyang

**Affiliations:** Department of Obstetrics and Gynecology, Shengjing Hospital of China Medical University, Shenyang, 110004 People’s Republic of China

**Keywords:** Statin, Ovarian cancer, Endometrial cancer

## Abstract

**Background:**

The relationship between statin use and the risk of ovarian or endometrial cancer remains controversial. Here, we investigated the relationship between statin use and the risk of ovarian and endometrial cancers.

**Methods:**

We conducted a meta-analysis using articles retrieved from the PubMed, Embase, and Web of Science databases. All original comparative studies published in English that were related to statin use and the risk of ovarian or endometrial cancer were included.

**Results:**

This meta-analysis included 19 studies enrolling 1,999,362 female subjects and 19,849 cancer cases (7,948 ovarian cancer cases and 11,901 endometrial cancer cases). The overall analysis indicated that statin use did not significantly reduce the risk of ovarian cancer [relative risk (RR) = 0.88, 95% confidence interval (CI) 0.76–1.03, *p* = 0.12] or the risk of endometrial cancer (RR = 0.88, 95% CI 0.78–1.00, *p* = 0.05.) Subgroup analyses based on study type, percentage of cancer cases, study location, and quality of studies also supported our conclusions. No association was found between long-term statin use (> 5 years) and the risk of ovarian cancer (RR = 0.73, 95% CI 0.51–1.04, *p* = 0.08) or endometrial cancer (RR = 0.79, 95% CI 0.58–1.08, *p* = 0.14).

**Conclusions:**

Statin use did not lower the risk of ovarian cancer or endometrial cancer. The long-term use of statins (> 5 years) was not associated with a reduction in the risk of ovarian or endometrial cancer.

**Electronic supplementary material:**

The online version of this article (10.1186/s12885-019-5954-0) contains supplementary material, which is available to authorized users.

## Background

Ovarian and endometrial cancers are the two most commonly diagnosed gynecological cancers, with the highest mortality of all gynecological cancers [1]. Ovarian cancer is the leading cause of cancer death in gynecological malignancies and the fifth leading cause of all cancers in women in the US [2]. Endometrial cancer is the most common gynecological malignancy in the US, with a death rate that has increased gradually [3].

Esposito et al. reported in a meta-analysis that metabolic syndrome is associated with an increased risk of ovarian and endometrial cancers [4]. Hydroxymethylglutaryl-CoA reductase inhibitors (statins) are cholesterol-lowering drugs that are primarily used for hypercholesterolemia therapy. They also have antitumor effects, which have been reported in experimental research on many cancers [5]. In recent years, the pharmacological functions of statins were found to be effective in several female reproductive diseases [6–9]. Statins inhibit 3-hydroxy-3-methylglutaryl coenzyme A (HMG CoA) reductase, the key enzyme in the mevalonate pathway for cholesterol synthesis. They can down regulate products of the mevalonate pathway, such as farnesyl diphosphate and geranyl diphosphate, which may contribute to regulating tumor cell growth, motility, and differentiation [10, 11].

Epidemiological studies of statins and the risk of endometrial or ovarian cancers have shown inconsistent results. A recent population-based study of 161,808 postmenopausal women indicated that statin use can reduce the risk of endometrial cancer but not that of ovarian cancer [12]. In contrast, another study reported that statin use reduced the risk of ovarian cancer [13]. Previous studies have reported that statin use was associated with a reduced risk of ovarian or endometrial cancer [14, 15].

Here, we conducted a meta-analysis to investigate the relationship between statin use and the risk of endocrine-related gynecological cancers.

## Methods

### Search strategy

This study was conducted in accordance with the Preferred Reporting Items for Systematic Reviews and Meta-Analyses (PRISMA) guidelines (http://www.prisma-statement.org/). A comprehensive search was performed of the PubMed, Embase, and Cochrane databases to August 1, 2018. The following MeSH and main keywords were used: “hydroxymethylglutaryl-CoA reductase inhibitor,” “Reductase Inhibitors, Hydroxymethylglutaryl-CoA,” “Inhibitors, HMG-CoA Reductase,” “Reductase Inhibitors, HMG-CoA,” “HMG-CoA Reductase Inhibitors,” “Statins, HMG-CoA,” “Statins,” “Hydroxymethylglutaryl-Coenzyme A Inhibitors,” and associated terms; and “Ovarian Neoplasm,” “Ovary Neoplasm,” “Ovary Cancer,” “Ovarian Cancer,” “Cancer of Ovary,” “Endometrial Neoplasm,” “Endometrial Carcinoma,” “Endometrial Cancer,” “Endometrium Cancer,” “Endometrium Carcinoma,” and associated terms. The language was restricted to English. For multiple-arm comparative studies, we extracted data only from the arms that matched our eligibility criteria. We also performed manual searches of the reference lists of the selected studies to retrieve all relevant data.

### Inclusion and exclusion criteria

Studies were selected according to the PICOS (population, intervention, comparison, outcomes, and study design) guidelines if they met the following inclusion criteria: (1) population: the entire population of women recruited to a certain trial, and the endpoint is incidence of ovarian or endometrial cancer; any histologic types of cancer were included for ovarian and endometrial cancers, including ovarian borderline malignancies; (2) intervention: patients used statins; (3) comparison: patients used statin versus control (placebo or no statin); (4) outcomes: studies compared morbidity between two groups using odds ratio (OR), relative risk (RR), or hazard ratio (HR) estimates with 95% confidence intervals (CIs), or provided sufficient data to compute morbidity; and (5) study design: studies were comparative [i.e., randomized control trials (RCTs) or observational studies].

Exclusion criteria were as follows: (1) population: mixed patients with other cancer types or patients with indeterminate cancer; (2) intervention: patients did not use statins; (3) comparison: patients used statin versus other drugs; (4) outcomes: studies lacked morbidity data; and (5) study design: single-arm study without a control group.

### Data extraction and quality assessment of included studies

Two reviewers (YW and FR) reviewed and assessed each of the included studies. Data extraction was performed independently. The other authors (ZS, PC, and SL) reviewed the included studies as well for inclusion. The following information was extracted from the included studies: first author, publication year, country of study, study type, cancer site and cases, number of subjects, percentage of cancer cases, study period, cases of statin use and control groups, follow-up time, and multivariable adjusted RR estimates with corresponding 95% CIs. The Jadad scale was used to assess the quality of the RCTs [16], and studies with 3 or more points were identified as high quality [17]. The Newcastle-Ottawa Scale (NOS) was used to assess the quality of the observational studies [18], and studies with 7 or more points were deemed high quality. Any disagreements were resolved by consensus between the two reviewers (YW and FR).

### Statistical analysis

This meta-analysis used the RR to estimate morbidity. All analyses used a random-effects model. The statistical values are reported with 95% CIs, and a two-tailed *p* < 0.05 was considered statistically significant. Heterogeneity among studies was tested by Cochran’s Q test (reported with a χ^2^ value and *p* value, with a *p* < 0.1 considered statistically significant) and the I^2^ statistic [19, 20]. I^2^ with values of 25, 50, and 75% demonstrated low, moderate, and high levels of heterogeneity, respectively [21]. We also conducted sensitivity analyses to assess the robustness of the results [22]. Subgroup analyses were conducted to explore sources of heterogeneity. Publication bias was assessed using Begg’s and Egger’s tests [23, 24]. All analyses were performed using Stata software version 12.0 (2011; Stata Corp., College Station, TX, USA).

## Results

### Characteristics of selected studies

**Fig. 1:**
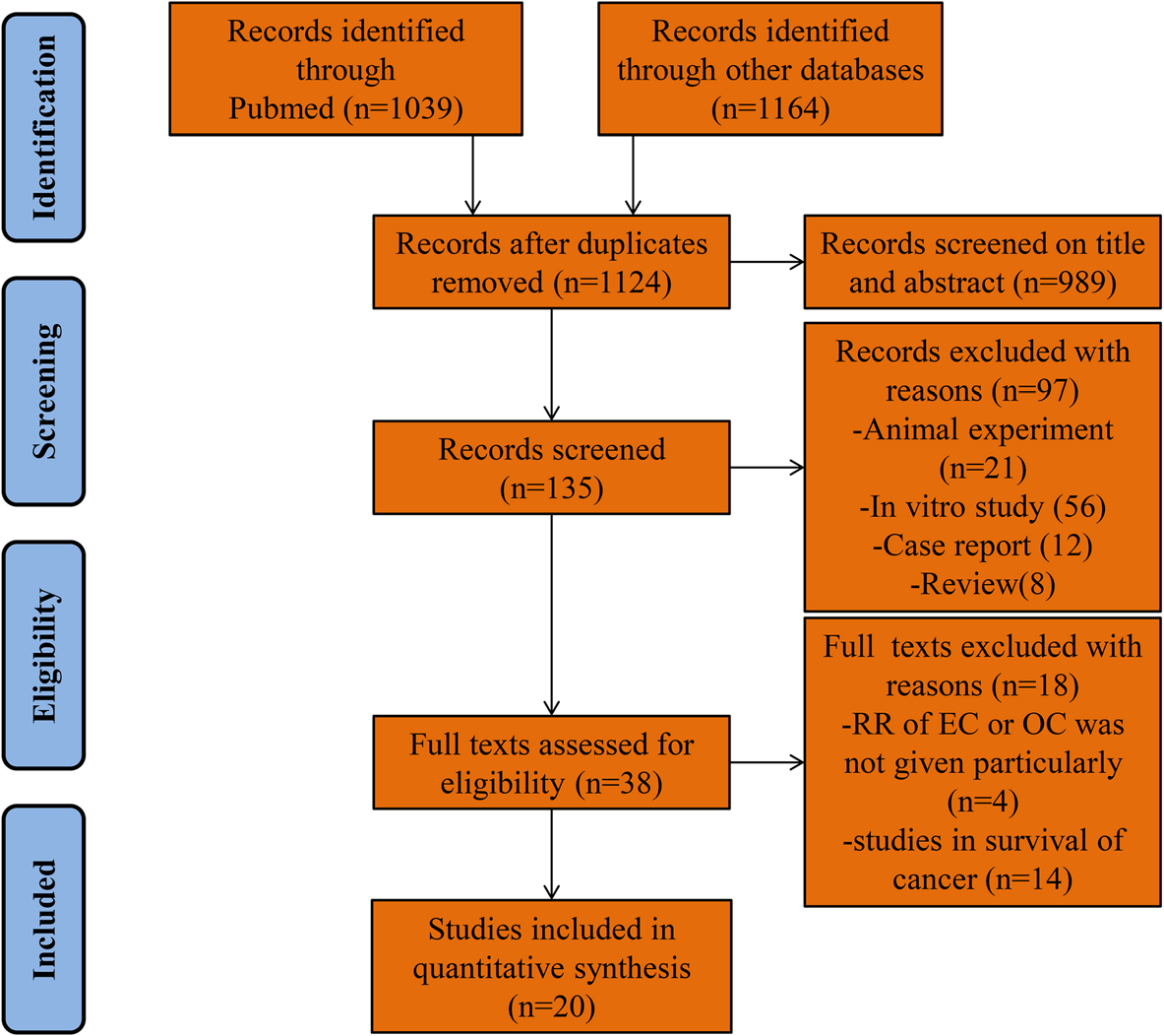
Flow chart of our systematic review and meta-analysis

A flow chart of the search strategy is illustrated in Fig. 1. The search retrieved 2,203 published studies. After removing duplicates, screening the title and abstract, and further evaluation, a total of 20 studies were included in our meta-analysis. Among these, one publication was excluded because it provided duplicated data [25]. The remaining 19 studies included two RCTs and 17 observational studies [12–15, 26–40]. Nine studies enrolled endometrial cancer patients, three enrolled ovarian cancer patients, and seven enrolled both. The cancer patients were from the population of women studied. The studies were carried out in eight countries, which included North America [12, 13, 26, 27, 29, 30, 32, 34, 39, 40], Europe [14, 15, 28, 31, 33, 35, 36, 38], and Asia [37]. Detailed information from the included studies is presented in Table 1.

**Table 1:** Baseline characteristics of the included studies

Study	Country	Study period	Study type	All female objects	EC			OC			EC		OC		Follow up (median/mean years)	NOS
					percentage of cancer cases	User	Nonuser	percentage of cancer cases	User	Nonuser	RR	95% CI	RR	95% CI		
Sperling 2017	Denmark	2000–2009	Case-control	77509	6.94%	610	4772	NA	NA	NA	1.03	0.94–1.14	NA	NA	NA	7
Urpilainen 2018	Finland	1996–2011	Case-control	748282	NA	NA	NA	0.04%	159	144	NA	NA	0.99	0.78–1.25	5.4	7
Jacobs 2011	USA	1997–2007	Cohort	73196	0.63%	134	327	NA	NA	NA	>5y:0.65 < 5y:1.11	0.45–0.94 0.84–1.46	NA	NA	NA	8
Friedman 2008	USA	1994–2003	Cohort	169261	0.12%	199	NA	0.05%	79	–	1.13	0.98–1.31	0.83	0.66–1.05	4.9	7
Kabat 2018	USA	1993–1998	Cohort	24208	0.48%	13	102	0.48%	15	100	0.96	0.54–1.7	1.24	0.72–2.12	NA	6
Leung 2013	China	2000–2008	Case-control	18055	1.23%	13	209	NA	NA	NA	0.43	0.20–0.93	NA	NA	4.2	7
Kaye 2004	UK	1990–2002	Case-control	8978	0.81%	3	70	1.01%	6	85	0.50	0.10–1.90	1.00	0.40–2.70	6.4	8
Haukka 2010	Finland	1996–2005	Cohort	473302	0.36%	836	885	NA	NA	NA	1.05	0.95–1.15	NA	NA	3	7
Fortuny 2009	USA	2001–2005	Case-control	936	50.11%	114	355	NA	NA	NA	1.30	0.81–2.10	NA	NA	NA	7
Baandrup2015	Denmark	2000–2011	Case-control	62809	NA	NA	NA	6.53%	434	3669	NA	NA	0.98	0.87–1.10	NA	8
Blais 2000	Canada	1988–1994	Case-control	4029	7.10%	26	260	NA	NA	NA	0.30	0.11–0.80	NA	NA	2.7	5
Clearfield 2001	USA	NA	RCT	997	0.40%	1	3	0.20%	0	2	0.33	0.03–3.19	0.20	0.01–4.15	5.2	6^a^
Lavie 2013	Israel	2003–2010	Case-control	682	31.52%	70	145	18.48%	38	88	0.55	0.37–0.81	0.49	0.28–0.81	NA	8
Yu 2009	USA	1990–2004	Cohort	73336	0.77%	18	550	0.44%	12	314	0.67	0.39–1.17	0.69	0.32–1.49	5.6	8
Coogan 2007	USA	1991–2005	Case-control	4641	4.59%	19	194	NA	NA	NA	1.30	0.70–2.40	NA	NA	NA	5
Desai 2018	USA	1993–1998	Cohort	161808	0.85%	78	1299	0.47%	62	701	0.74	0.59–0.94	1.15	0.89–1.50	10.8	8
Arima 2017	Finland	1996–2011	Case-control	92366	0.64%	270	320	NA	NA	NA	0.78	0.65–0.94	NA	NA	5.5	7
Strandberg 2004	Nordic countries	1988–1999	RCT	827	0.73%	3	3	NA	NA	NA	1.03	0.21–5.14	NA	NA	10.4	5^a^
Akinwunmi 2018	USA	1992–2008	Case-control	4140	NA	NA	NA	49.28%	188	1852	NA	NA	0.68	0.54–0.85	NA	8

*RCT* randomized controlled trial, *NOS* Newcastle–Ottawa Scale, *NA* not applicable

^a^Jadad scale was used to assess the quality of the randomized clinical trials

### Risk of ovarian cancer among statin users

**Fig. 2:**
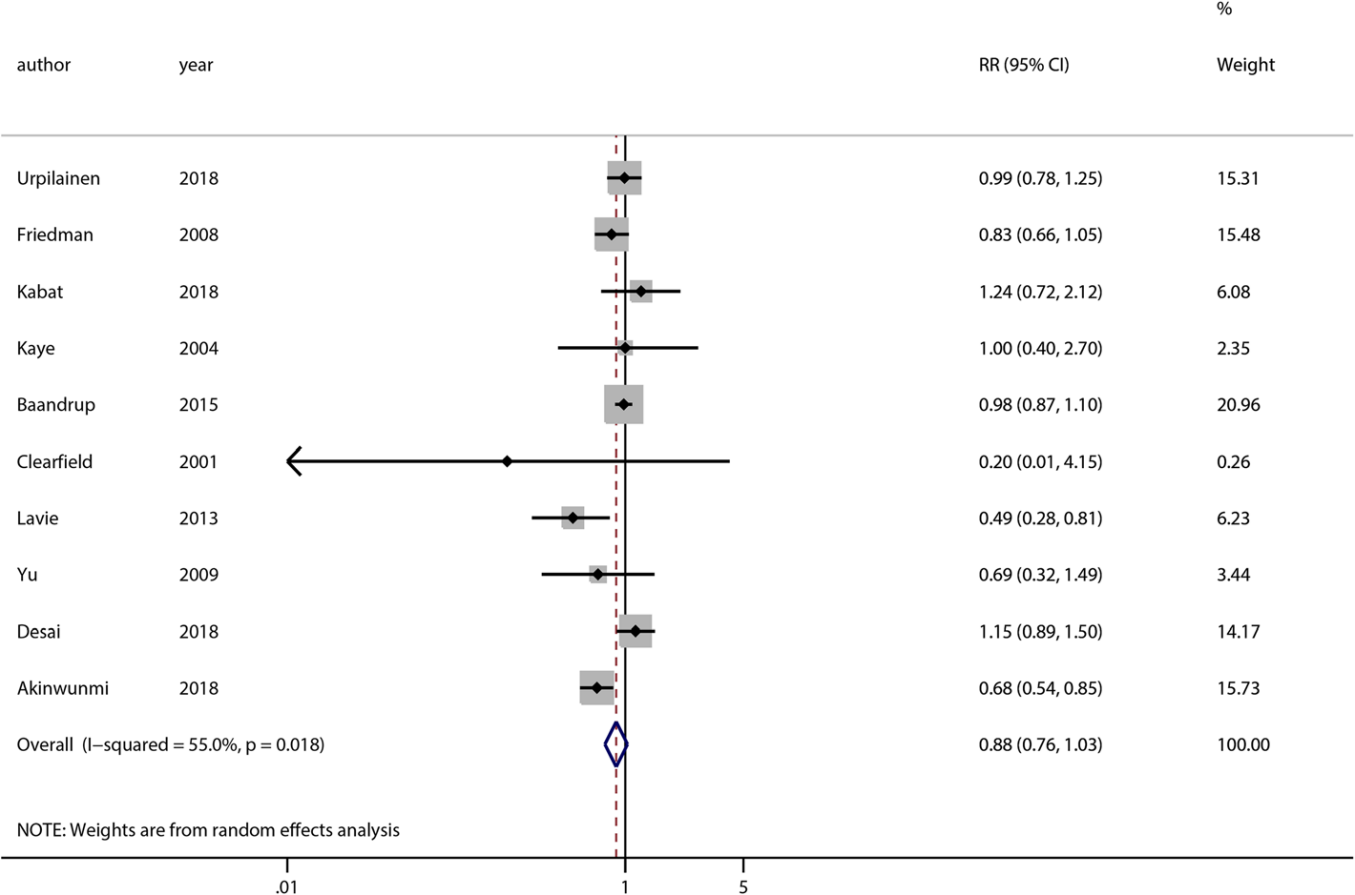
Overall analysis of statin use and the risk of ovarian cancer

The meta-analysis included 10 studies of ovarian cancer. Our pooled analysis indicated that statin use did not reduce the risk of ovarian cancer (RR = 0.88, 95% CI = 0.76–1.03, *p* = 0.12) (Fig. 2). A moderately significant heterogeneity was observed among the studies (χ^2^ = 20.00, *p* = 0.02, I^2^ = 55.0%). Sensitivity analyses were carried out by excluding studies one-by-one, and we found that the corresponding pooled RRs were not significantly altered, which affirmed the robustness of the result (Additional file 1: Figure S1**).** We conducted subgroup analyses of statin use and the risk of ovarian cancer based on study type, percentage of cancer cases, geographical study location (continent), and quality of studies. There was no significant association between statin use and the risk of ovarian cancer in the RCTs (RR = 0.20, 95% CI = 0.01–4.07, *p* = 0.30), cohort studies (RR = 0.98, 95% CI = 0.77–1.24, *p* = 0.85), or case-control studies (RR = 0.82, 95% CI = 0.65–1.04, *p* = 0.10). Subgroup analysis based on the percentage of cancer cases indicated that there was no significant association in groups with ≥1% (RR = 0.76, 95% CI = 0.55–1.05, *p* = 0.10) or < 1% (RR = 0.97, 95% CI = 0.83–1.14, *p* = 0.74). Subgroup analysis of study location showed no reduced risk of ovarian cancer among statin users in North America (RR =0.88, 95% CI = 0.69–1.12, *p* = 0.29) or Europe (RR =0.90, 95% CI = 0.72–1.12, *p* = 0.34). Additionally, no decreased risk of ovarian cancer in statin users was observed, whether studies were high quality or low quality (Table 2). There was no evidence of publication bias (Begg’s test, *p* = 0.86, [Additional file 2: Figure S2A]; Egger’s test, *p* = 0.34, [Additional file 2: Figure S2B]).

**Table 2:** Subgroup analyses of statin use and risk of endocrine-related gynecologic cancers

Characteristics	Ovarian Cancer				Endometrial Cancer			
	Study number	RR(95%CI)	*P* value	Heterogeneity	Study number	RR(95% CI)	*P* value	Heterogeneity
Study type	10	0.88 (0.76,1.03)	0.12	55.0%	16	0.88 (0.78,1.00)	0.05	66.3%
RCT	1	0.20 (0.01,4.07)	0.30	0.0%	2	0.72 (0.19,2.68)	0.62	0.0%
Cohort	4	0.98 (0.77,1.24)	0.85	38.9%	7	0.93 (0.80,1.10)	0.38	66.5%
Case-control	5	0.82 (0.65,1.04)	0.10	70.6%	7	0.80 (0.62,1.03)	0.09	74.1%
Percentage of cancer cases								
≥ 1%	4	0.76 (0.55,1.05)	0.10	76.8%	6	0.79 (0.54,1.15)	0.21	76.5%
< 1%	6	0.97 (0.83,1.14)	0.74	16.6%	10	0.90 (0.78,1.04)	0.14	61.8%
Study location								
North America	6	0.88 (0.69,1.12)	0.29	58.1%	9	0.90 (0.73,1.11)	0.31	64.2%
Europe	4	0.90 (0.72,1.12)	0.34	52.7%	6	0.89 (0.75,1.05)	0.18	72.2%
Asia	0	–	–	–	1	0.43 (0.20,0.94)	0.03	0.0%
Quality of studies								
High	9	0.86 (0.74,1.01)	0.07	57.4%	13	0.88 (0.78,1.01)	0.06	68.5%
Low	1	1.24 (0.72,2.12)	0.44	0.0%	3	0.80 (0.39,1.62)	0.53	67.4%
Dduration of statin use								
long-term use	5	0.73 (0.51,1.04)	0.08	58.9%	6	0.79 (0.58,1.08)	0.14	58.2%
Study in diabetics	1	0.96 (0.75,1.23)	0.75	0.0%	1	0.78 (0.65,0.94)	0.01	0.0%

### Risk of endometrial cancer among statin users

**Fig. 3:**
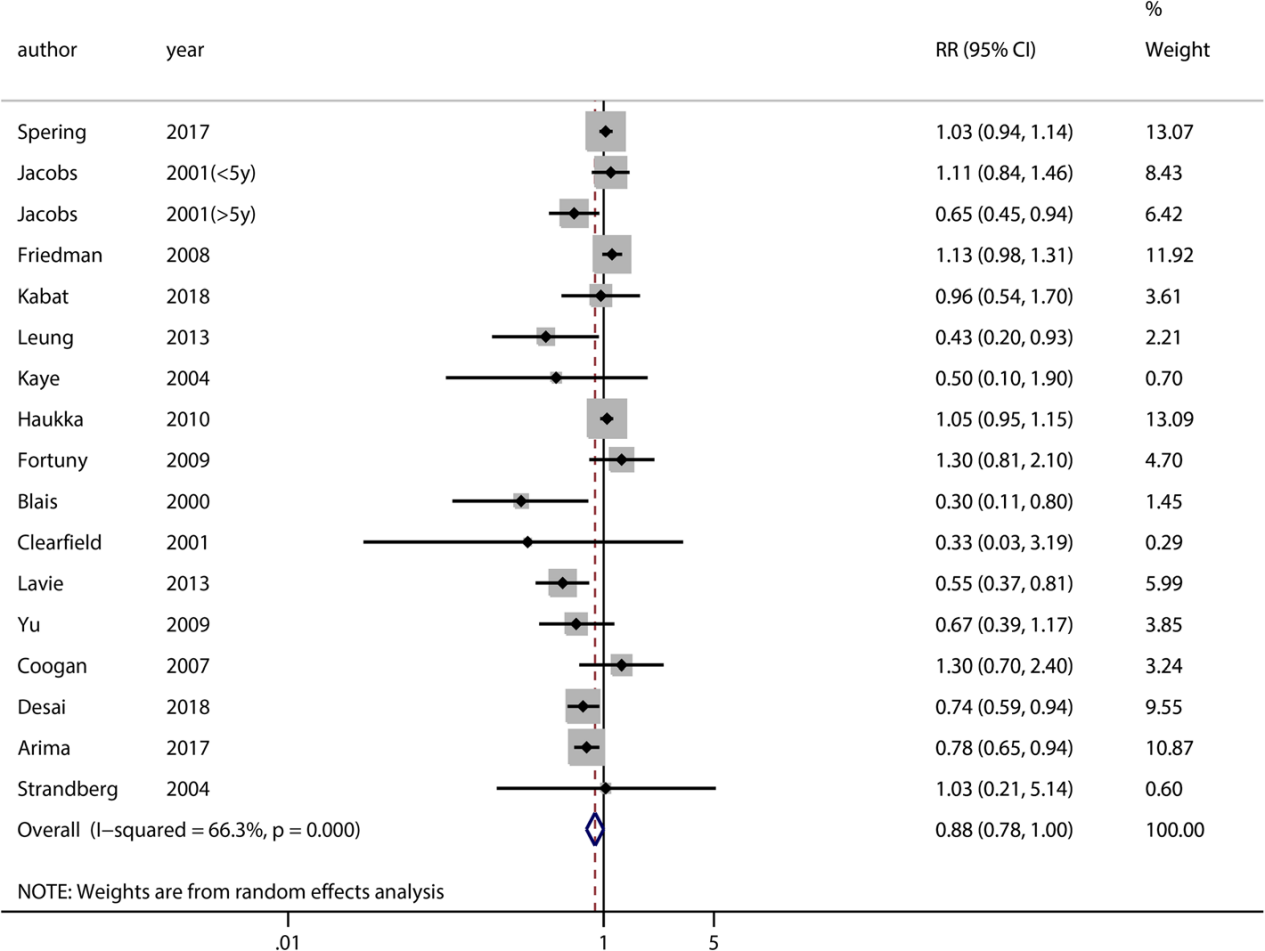
Overall analysis of statin use and the risk of endometrial cancer

The meta-analysis included 16 studies of endometrial cancer. There was no significant association between the use of statins and the risk of endometrial cancer (RR = 0.88, 95% CI = 0.78–1.00, *p* = 0.05) (Fig. 3). A moderately significant heterogeneity was observed among the studies (χ^2^ = 47.50, *P* = 0.00, I^2^ = 66.3%). Sensitivity analyses were carried out by excluding studies one-by-one, and we found that six studies [15, 26, 30, 32, 34, 35] seemed to affect the heterogeneity. Excluding any one of these studies from the overall analysis resulted in statistically significant results, but the upper limits of the 95% confidence interval were approximately 1.00 (Additional file 3: Figure S3). We also conducted subgroup analyses by study type, percentage of cancer cases, study location, and quality of studies. There was no significant association between statin use and the risk of endometrial cancer in the RCTs (RR = 0.72, 95% CI = 0.19–2.68, *p* = 0.62), cohort studies (RR = 0.93, 95% CI = 0.80–1.10, *p* = 0.38), or case-control studies (RR = 0.80, 95% CI = 0.62–1.03, *p* = 0.09). Subgroup analysis based on the percentage of cancer cases indicated that there was no significant association in groups with ≥1% (RR = 0.79, 95% CI = 0.54–1.15, *p* = 0.21) or < 1% (RR = 0.90, 95% CI = 0.78–1.04, *p* = 0.14) of cancer cases in the study population. Subgroup analysis of studies based on geographical location showed a significantly reduced risk of endometrial cancer among statin users in Asia (RR = 0.43, 95% CI = 0.20–0.94, *p* = 0.03), but none in North America (RR = 0.90, 95% CI = 0.73–1.11, *p* = 0.31) or Europe (RR = 0.89, 95% CI = 0.75–1.05, *p* = 0.18). Again, no significant association between statin use and risk of endometrial cancer was observed in studies of high quality or low quality (Table 2). There was evidence of potential publication bias (Begg’s test, *P* = 0.434, [Additional file 4: Figure S4A]; Egger’s test, *p* = 0.03, [Additional file 4: Figure S4B]).

### Long-term statin use and the risk of ovarian or endometrial cancer

**Fig. 4:**
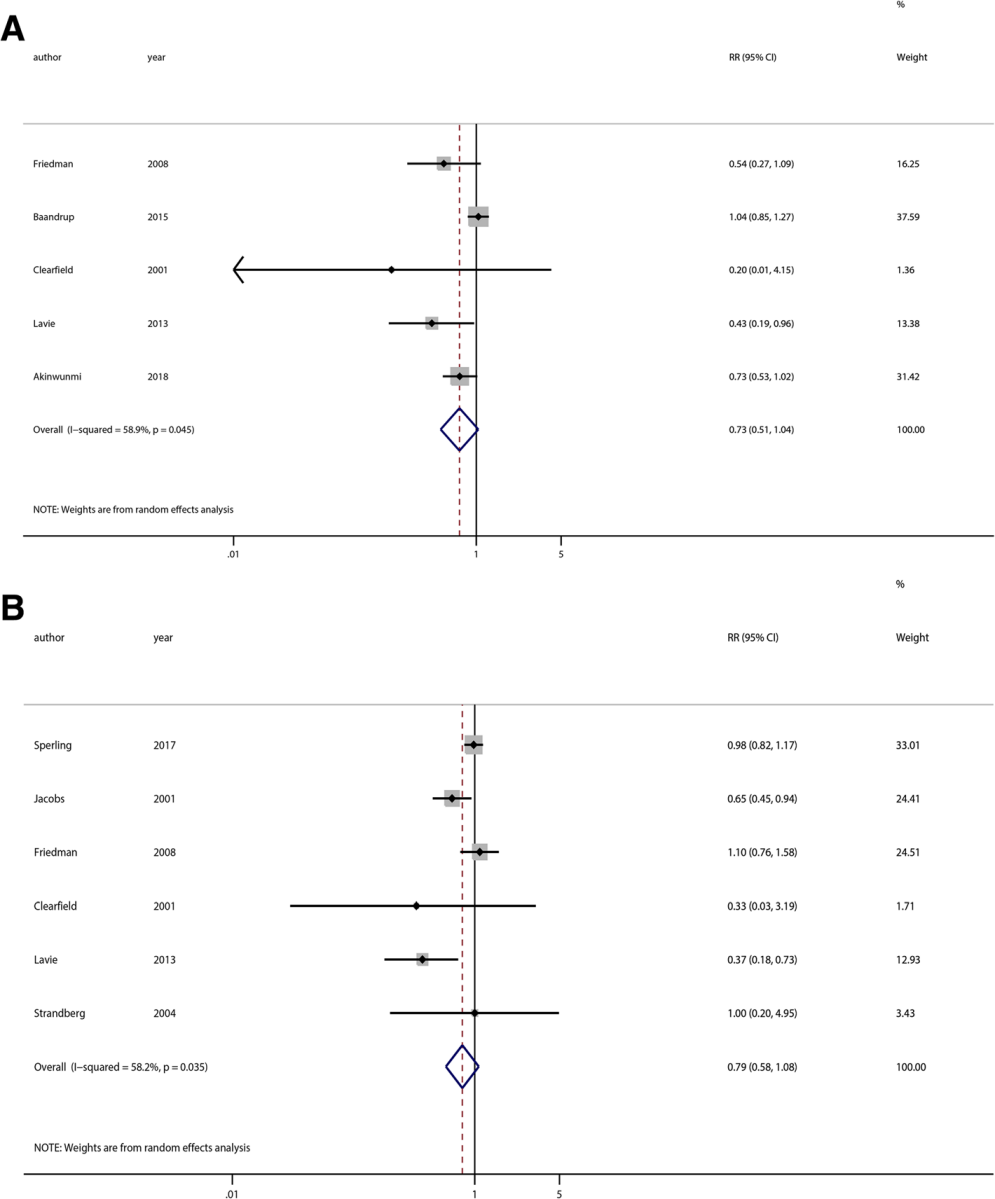
Subgroup analyses of long-term statin use and the risk of ovarian and endometrial cancer. **a** Ovarian cancer; **b** Endometrial cancer

The meta-analysis included five studies of ovarian cancer [13, 14, 30, 36, 39] and six studies of endometrial cancer [15, 30, 33, 34, 36, 39] that analyzed long-term statin use (> 5 years). There was no association between long-term statin use (> 5 years) and the risk of endometrial cancer (RR = 0.79, 95% CI = 0.58–1.08, *p* = 0.14) or ovarian cancer (RR = 0.73, 95% CI = 0.51–1.04, *p* = 0.08) (Fig. 4).

## Discussion

The association between statin use and the risk of endometrial or ovarian cancer remains controversial. Sperling et al. [15] reported no association between statin use and the risk of endometrial cancer, whereas Desai et al. [12] reported that statins reduced the risk of endometrial cancer. Some studies have reported a significant association between statin use and the risk of ovarian cancer [13, 25, 36], but others found no such association [12, 31]. In 2014, Liu et al. [41] performed a meta-analysis of the effect of statin use on the risk of gynecological cancers and reported that statin use lowered the risk of ovarian cancer but not that of endometrial cancer. The debate has continued after that publication, and the results of more recent studies have also been inconsistent.

Our meta-analysis included 19 studies enrolling 1,999,362 women. Seven of these studies were published after the last meta-analysis in 2014 and included an additional 1,171,122 subjects. Our pooled analysis indicated that statin use did not reduce the risk of ovarian or endometrial cancer. In addition, we conducted subgroup analyses by study type, percentage of cancer cases, quality of studies, and study location to explore sources of heterogeneity. Two RCTs, six cohort studies and 11 case-control studies were included, and there was no significant association between the use of statins and the risk of ovarian or endometrial cancer based on study type. We used the Jadad scale and NOS criterion to assess the quality of the RCTs and observational studies, respectively. The two RCTs were deemed to be of high quality, with Jadad total scores ≥3, and 14 observational studies had NOS scores ≥7, identifying them as high quality. Subgroup analyses based on the quality of studies indicated that no significant association between statin use and the risk of endometrial cancer was observed in studies of high quality or low quality. The studies we included had different numbers of participants, most included large populations, and the percentage of cancer patients was low and varied. However, we found no significant differences in the association of statin use and the risk of ovarian or endometrial cancer in groups with percentages of cancer cases ≥1% versus groups with < 1%. All of the above subgroup analyses supported our pooled conclusions. In the subgroup analyses based on study location, our results showed a similar tendency, with no reduced risk of ovarian or endometrial cancer in statin users, although a subgroup of statin users in Asia did have a significantly reduced risk of endometrial cancer. This result might be due to regional disparities or racial differences. People in different countries have different dietary habits. Patel et al. reported that metabolic syndrome was less prevalent in Asian populations than it is in US populations [42]. Therefore, the effects of statin use may vary according to location. However, this meta-analysis subgroup (endometrial cancer patients in Asia) included only one study conducted in Asia, which enrolled fewer subjects than in other studies. Hence, the effects of statin use in endometrial cancer patients require further exploration, and our results require validation from additional RCTs enrolling large populations.

Ovarian and endometrial cancers may be associated with diabetes mellitus. Previous studies reported that patients with diabetes mellitus had an increased risk of ovarian cancer, and patients with ovarian cancer and diabetes had poor survival [43, 44]. The incidence of endometrial cancer is increasing due to global increases in diabetes and obesity [45, 46], suggesting that we should target women with diabetes. We planned to conduct subgroup analyses of studies enrolling women with diabetes, but the number of studies was limited. Although the risk of endometrial cancer was significantly reduced in statin users, the results require validation with additional studies enrolling larger populations.

Studies investigating the effects of long-term statin use on ovarian or endometrial cancer have reported inconsistent results. Some studies found no association [34]. In contrast, the meta-analysis of Liu et al. [41] reported that long-term statin use significantly reduced the risk of ovarian cancer. Our subgroup analyses of long-term statin use included three more studies and 5,817 additional subjects than the previous meta-analysis [41] and showed no significant association between long-term statin use and the risk of ovarian or endometrial cancer. As we found no reduction in the risk of ovarian or endometrial cancer in statin users, long-term statin use also had no protective effect. The majority of studies of the effect of long-term statin use indicated no reduced risk of ovarian or endometrial cancer in statin users. Indeed, a recent observational study by Desai et al. [12] reported that the use of pravastatin was associated with an increased risk of ovarian cancer. This study enrolled a large number of female subjects (161,808) and investigated the relationship between different drug types and cancer risk. Thus, the long-term effect of statins must still be considered unanswered, and more RCTs or high-quality observational studies are needed to verify the effects of long-term statin use on ovarian or endometrial cancer.

Our study has some advantages. The meta-analysis included 19 studies enrolling 1,999,362 female subjects and 19,849 cancer cases. Our inclusion and exclusion criteria were based on PICOS criteria. The Jadad scale was used to assess the quality of the RCTs, and the NOS criterion was used to evaluate the quality of the observational studies; most of the studies were high quality. The 2014 meta-analysis [41] investigated the association between statin use and the risk of gynecological cancers. In contrast, we investigated the association between statin use and the risk of ovarian and endometrial cancers separately. Our meta-analysis included seven more studies and a larger number of participants than the previous meta-analysis, and our results differed from those of the earlier study. Our subgroup analyses based on study type, percentage of cancer cases, study location, and quality of studies largely supported our conclusions.

There were some limitations in our meta-analysis, which will be resolved in future studies. First, in the selected studies, women were prescribed statins primarily to treat metabolic syndrome, which may independently induce ovarian or endometrial cancer. As a result, it was not possible to pool metabolic status for the observational studies included in this meta-analysis. Moreover, in some studies, patients were prescribed other medications in addition to statins. As other medications may be associated with the risk of cancer, for example, the use of metformin or oral contraceptives may lower the risk of gynecologic cancer [47, 48], and hormone replacement therapy may influence the risk of gynecologic cancer [49], there was the possibility that our results had bias, which could be of concern. Hence, to confirm the conclusions of this meta-analysis, further RCTs enrolling larger populations to investigate the association between statin use and the risk of ovarian or endometrial cancer are needed.

Second, the heterogeneity of our study was significant. Our study included 2 RCTs and 17 observational studies; the two RCTs included 12 total cancer cases, whereas the largest studies included approximately 5,000 cancer cases [15]. Among the included observational studies, the study by Lavie et al. [36] is a case-control trial in which controls were individually matched to cases according to age, sex, clinic and ethnic group. Therefore, the study excluded many confounding factors, which might lead to a more reliable result. As it has been reported that statin use could reduce the risk of endometrial and ovarian cancers, we cannot ignore the important finding. Although our pooled analysis was negative, the majority of the studies we included were retrospective, which may contain selection, information, and confounding bias. More RCTs or high-quality observational studies are needed to confirm or update our meta-analysis. In addition, we extracted RR data directly or indirectly to represent the relationship between statin use and ovarian or endometrial cancer. However, each of the included studies utilized different study designs according to the different statins or different statin doses. Most studies observed the outcomes of overall statin use (rather than that of a particular drug) and cancer risk. Other studies classified statins as lipophilic or hydrophilic [13, 37] or separately observed different drugs [15, 33, 35, 39]. As different statins have different half-lives, different types or doses of statin have been proposed to contribute differently to clinical outcomes. However, the results could not be combined because of such differences in the included studies. Most studies did not record the duration of statin use, and other studies evaluating statin duration were not coincident in the observation time. Thus, we could not combine the results according to the duration of statin use specifically, but only extracted data for long-term statin use (> 5 years). Moreover, the studies we included differed in terms of research methods, follow-up time, and source of the population; any of these factors may have induced study heterogeneity.

Third, the sensitivity analysis indicated that excluding studies one-by-one did not alter the corresponding pooled RRs in the ovarian cancer group, which affirmed the robustness of the result. However, for the studies of endometrial cancer, we found that six studies seemed to affect the heterogeneity. When we excluded any one of these six studies from the overall analysis, statistically significant results were obtained. However, the upper limits of the 95% confidence intervals were approximately 1.00. Thus, as we did not think that excluding so many studies was appropriate, we maintained the original pooled results. In addition, when we conducted analyses of statin use and the risk of ovarian cancer, there was no evidence of publication bias. However, in the overall analyses of endometrial cancer, although there was no significance in Begg’s test, Egger’s test was statistically significant, which indicated a potential publication bias. The most likely explanation is the preferential publication of positive findings.

Fourth, the number of RCTs investigating statin use and the risk of ovarian or endometrial cancer was limited. The search retrieved two RCTs with 12 cancer cases but 17 observational studies with 19,837 cancer cases. Moreover, in these RCTs, statins were used to treat patients with coronary heart disease or hyperlipemia. Cancer was not a primary outcome. Hence, the results from the two RCTs were not powerful enough to conclude the outcome of cancers. Thus, further RCTs investigating statin use and the risk of ovarian or endometrial cancer will be required to confirm these results.

## Conclusions

This meta-analysis identified no reduction in the risk of ovarian or endometrial cancer for patients who used statins. In addition, the long-term use of statins (> 5 years) was not associated with a reduction in the risk of ovarian or endometrial cancer. To confirm the conclusions of this meta-analysis, further RCTs enrolling larger populations to investigate the association between statin use and the risk of ovarian or endometrial cancer are needed.

## Additional files

Additional file 1: **Figure S1.** Sensitivity analyses based on statin use and the risk of ovarian cancer. (TIF 1190 kb)

Additional file 2: **Figure S2.** Publication bias in terms of statin use and the risk of ovarian cancer. (A) Begg’s test; (B) Egger’s test. (TIF 1616 kb)

Additional file 3: **Figure S3.** Sensitivity analyses based on statin use and the risk of endometrial cancer. (TIF 1344 kb)

Additional file 4: **Figure S4.** Publication bias in terms of statin use and the risk of endometrial cancer. (A) Begg’s test; (B) Egger’s test. (TIF 1658 kb)

## Data Availability

Input data for the analyses are available from the corresponding author on request.

## Abbreviations

CI: Confidence interval
HMG CoA: 3-hydroxy-3-methylglutaryl coenzyme A
HR: Hazard ratio
NA: Not applicable
NOS: Newcastle-Ottawa Scale
OR: Odds ratio
PICOS: Population, intervention, comparison, outcomes, and study design
PRISMA: Preferred Reporting Items for Systematic Reviews and Meta-Analyses
RCT: Randomized control trial
RR: Relative risk
Statins: Hydroxymethylglutaryl-CoA reductase inhibitors
