# Supplementary figures and images for: Statin use and the risk of ovarian and endometrial cancers: a meta-analysis
Source: BMC Cancer. 2019 Jul 24;19:730. doi: 10.1186/s12885-019-5954-0 (PMC6657066; doi:10.1186/s12885-019-5954-0)

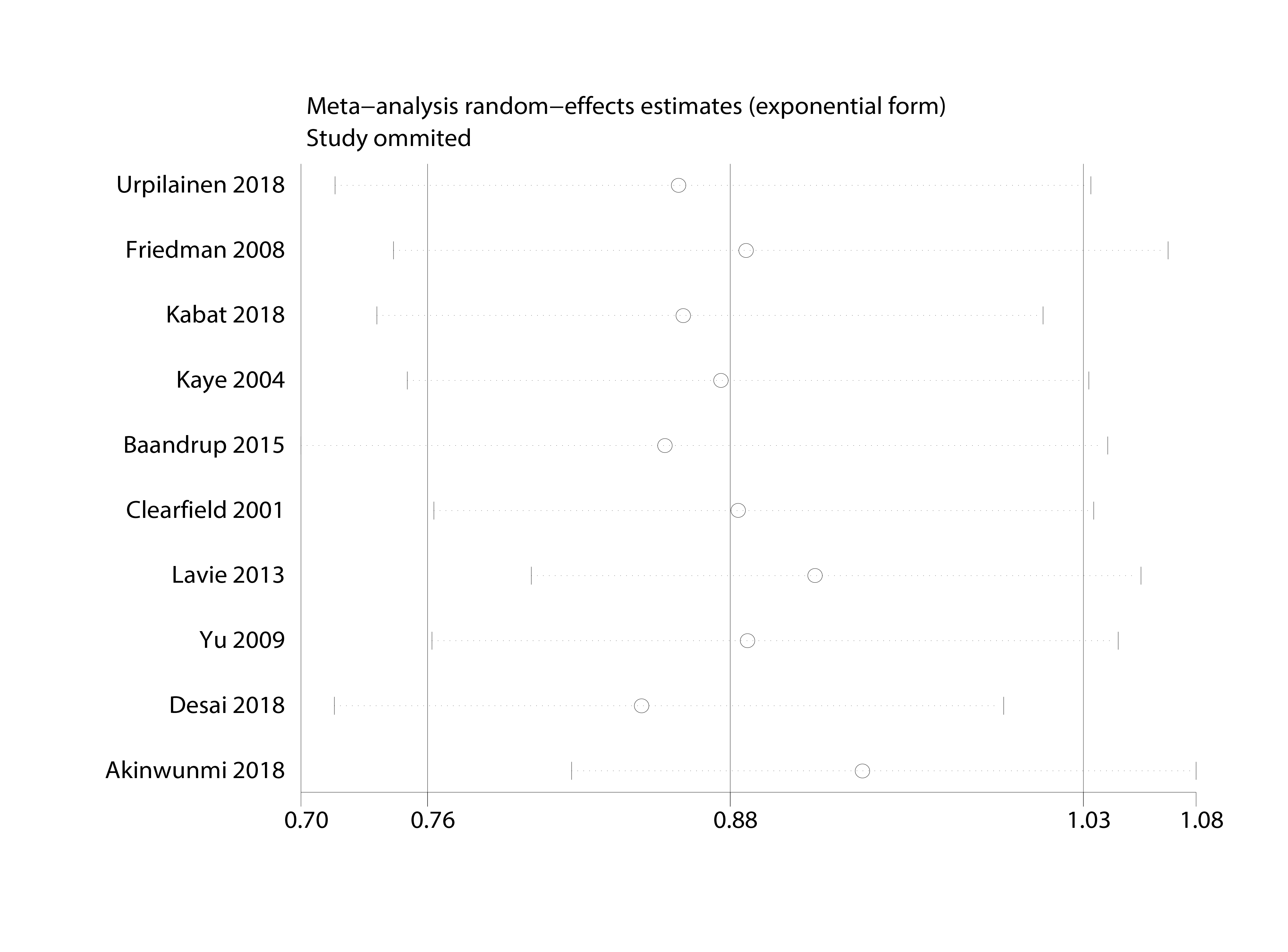

Supplement: Supplementary file 1 — Figure S1. Sensitivity analyses based on statin use and the risk of ovarian cancer. (TIF 1190 kb) [file 12885_2019_5954_MOESM1_ESM.tif]

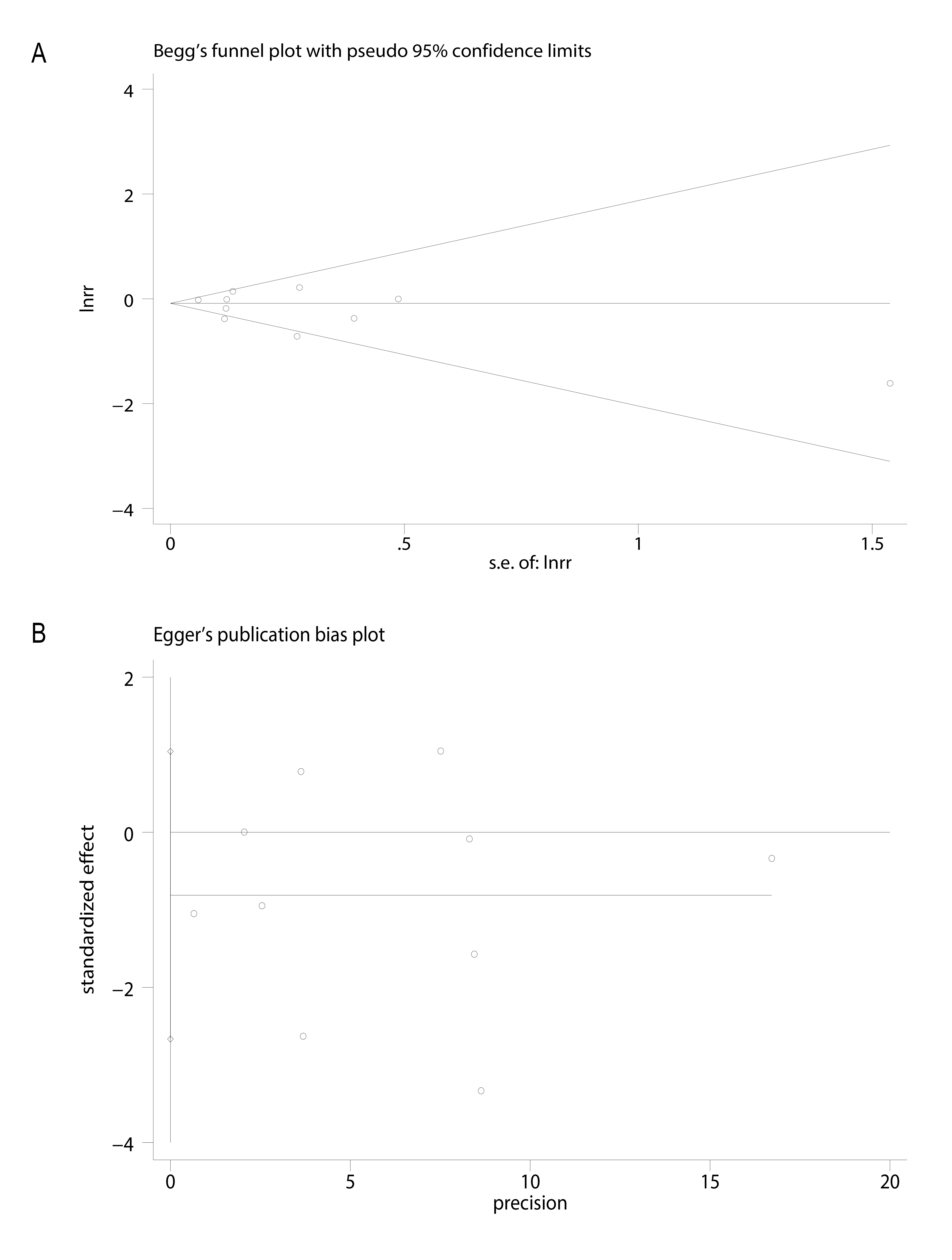

Supplement: Supplementary file 2 — Figure S2. Publication bias in terms of statin use and the risk of ovarian cancer. (A) Begg’s test; (B) Egger’s test. (TIF 1616 kb) [file 12885_2019_5954_MOESM2_ESM.tif]

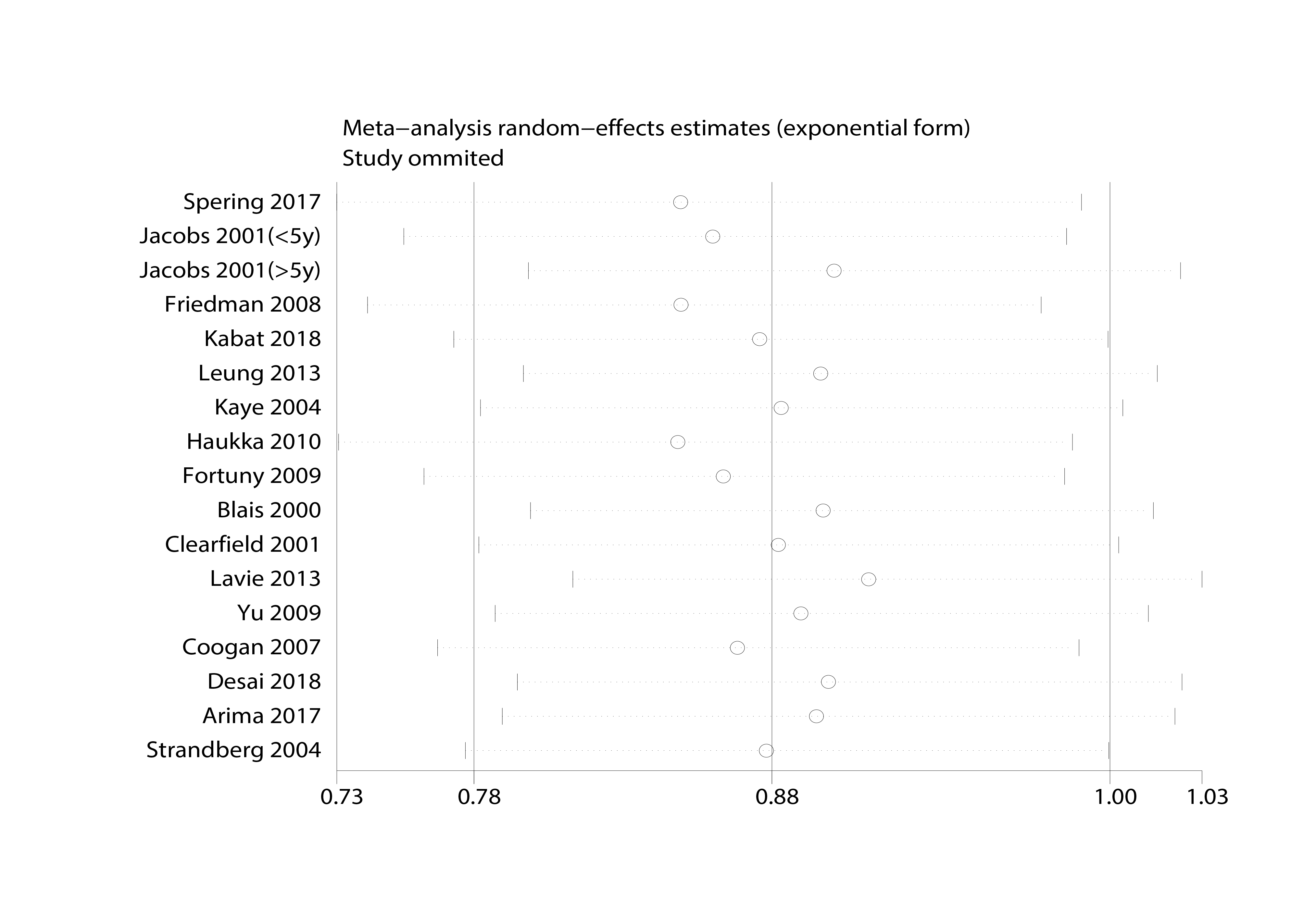

Supplement: Supplementary file 3 — Figure S3. Sensitivity analyses based on statin use and the risk of endometrial cancer. (TIF 1344 kb) [file 12885_2019_5954_MOESM3_ESM.tif]

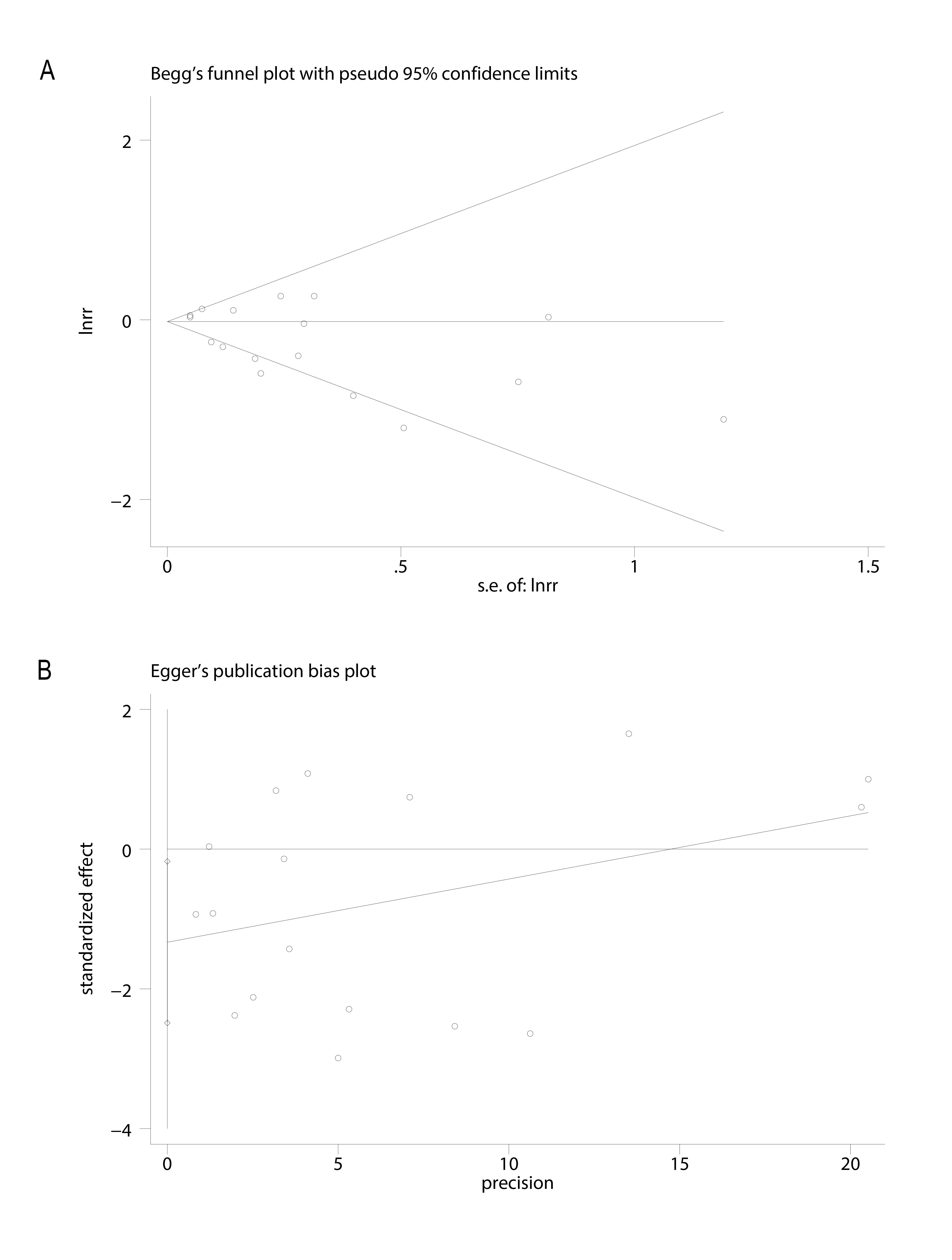

Supplement: Supplementary file 4 — Figure S4. Publication bias in terms of statin use and the risk of endometrial cancer. (A) Begg’s test; (B) Egger’s test. (TIF 1658 kb) [file 12885_2019_5954_MOESM4_ESM.tif]
